# Trophic transfer of biodiversity effects: functional equivalence of prey diversity and enrichment?

**DOI:** 10.1002/ece3.415

**Published:** 2012-11-08

**Authors:** Stephan Behl, Vera Schryver, Sebastian Diehl, Herwig Stibor

**Affiliations:** 1 Department Biology II, Aquatic Ecology, Ludwig-Maximilians-Universität MünchenGroßhaderner Straße 2, 82152, Planegg-Martinsried, Germany; 2 European Institute for Marine Studies, Place Nicolas Copernic, Technopôle Brest-Iroise29280, Plouzané, France; 3 Department of Ecology and Environmental Science, Umeå University90187, Umeå, Sweden

**Keywords:** Biodiversity, carbon-to-phosphorus ratio, chlorophytes, *Daphnia magna*, enrichment, light intensity, niche complementarity, phytoplankton, resource use efficiency, trophic transfer

## Abstract

Producer diversity is frequently assumed to be detrimental to herbivores, because less edible taxa are more likely to dominate diverse communities. Many producers are, however, complementary in their resource use, and primary production is often positively related to producer diversity. We performed an experiment with microalgae and a generalist herbivore to explore the hypothesis that such positive effects are transferred up the food chain and are functionally comparable to effects of enrichment with a limiting resource. In both absence and presence of grazers, primary production was positively affected by both light supply and producer diversity. Survival, reproduction, and biomass of herbivores were also positively affected by light supply and producer diversity, with both factors contributing equally to grazer performance. We conclude that producer diversity can indeed have similar positive effects on secondary production as enrichment with a limiting resource and discuss conditions under which such positive effects are likely to dominate over negative ones.

## Introduction

Trophic transfer of primary production is a conceptual cornerstone of ecosystem ecology. Numerous studies have demonstrated that increased primary production usually translates into increased secondary production. Historically, ecologists have focused on enrichment (increased supply with limiting resources) as the source of increased primary production, and a large body of ecological theory has been developed to describe the consequences of enrichment for ecological communities (Oksanen et al. 1981; Holt et al. 1994; Grover 1995; Wollrab et al. 2012). More recently, it has been discovered that primary production is also positively related to producer diversity, a major underlying mechanism being niche complementarity and, consequently, more efficient use of limiting resources by primary producers (van Ruijven and Berendse 2005; Striebel et al. 2009a; Cardinale 2011). Positive effects of diversity on primary production can be on the same order as effects of enrichment (Reich et al. 2001; Fridley 2003; Hooper et al. 2012). This raises the question: Are resource supply and diversity of primary producers functionally equivalent in that their positive effects on primary production are transferred up the food chain? Although studies of biodiversity effects spanning more than one trophic level are receiving increasing attention (Duffy et al. 2007; Srivastava et al. 2009), this hypothesis has, to our knowledge, not yet been clearly formulated and addressed with experiments.

Experimental tests of this hypothesis must fulfill at least three conditions. First, enrichment with a limiting resource and producer diversity must be manipulated independently. Second, primary production must respond positively to each of these factors in isolation to enable a comparison of their relative impacts on secondary production. Finally, only a single species of a generalist herbivore should be present in the system. With several herbivore species, it would be difficult to separate effects of producer productivity from effects of herbivore diversity (e.g., synergistic or compensatory responses among different herbivores). Although numerous experimental studies have examined diversity effects in producer–consumer systems (e.g., Siemann et al. 1998; Koricheva et al. 2000; Norberg 2000; Steiner 2001; Fox 2004; Gamfeldt et al. 2005; Bruno et al. 2008; Dzialowski and Smith 2008; Narvani and Mazumder 2010), most of them focused on trophic consequences of consumer diversity (or on joint effects of producer and consumer diversity), and none of them tried to compare effect magnitudes of diversity with effect magnitudes of resource enrichment.

Enrichment and trophic transfer of primary production have been particularly well studied in freshwater systems. For example, comparative studies have revealed positive relationships between nutrient enrichment and biomass at all trophic levels, the strengths of these relationships being modulated by food-web structure (Hanson and Peters 1984; Persson et al. 1992). More recently, experimental and comparative studies have explored light limitation in lakes. Although these studies found positive relationships between light supply and both primary and secondary production (Diehl et al. 2005; Berger et al. 2006; Karlsson et al. 2009), enrichment with light is conceptually different from nutrient enrichment. Specifically, light supply depends not only on incident radiation but also on physical properties such as water depth and background attenuation (Huisman and Weissing 1994). As a consequence, light and nutrients are often limiting in different parts of the water column (Yoshiyama et al. 2009). Increased carbon fixation at higher light supply therefore tends to increase nutrient use efficiency, which is expressed in an enhanced carbon-to-nutrient ratio of algal biomass (Sterner et al. 1997; Diehl et al. 2005; Berger et al. 2006).

Light differs from mineral resources also in that it is always supplied in a vertical gradient of decreasing intensity and changing spectral quality. Hence, planktonic algae experience large fluctuations in the quantity and quality of light when vertically mixed (Ferris and Christian 1991). The resulting unpredictable shifts in light supply and spectral composition plausibly explain the high diversity of photosynthetic pigments in the phytoplankton (Falkowski et al. 2004). Pigment composition is thus a trait characterizing the spectral niche of an algal taxon (Stomp et al. 2004), and spectrally more diverse phytoplankton communities should harvest light more efficiently. Such patterns have indeed been observed; that is, more diverse phytoplankton communities show higher pigment diversity, absorb a higher fraction of available light, and fix more carbon (Striebel et al. 2009a; Behl et al. 2011). Interestingly, experiments with natural communities have shown that both light enrichment and increased phytoplankton diversity independently increase nutrient use efficiency, that is, yield higher carbon-to-nutrient ratios of algal biomass (Dickman et al. 2006; Striebel et al. 2008). These observations support the hypothesis that increased phytoplankton diversity and light enrichment have similar effects on phytoplankton production and we conjecture that these effects should be similarly transferred to herbivores.

Here, we describe a laboratory experiment comparing the effects of light enrichment and algal producer diversity on survival, reproduction, and biomass of a generalist grazer. We first review a couple of earlier experiments investigating the separate effects of light supply and producer diversity on primary production in absence of grazers. These experiments were conducted with the same algal taxa and under similar conditions as the grazer experiment. We then present the results of the grazer experiment with a focus on comparing the relative contributions of light supply and producer diversity to grazer performance. We found that, in both absence and presence of grazers, primary production was positively related to both light supply and producer diversity. Survival, reproduction, and biomass of herbivores were also all positively related to light supply and producer diversity, with both factors contributing about equally to grazer performance. We conclude that producer diversity can have a similarly strong, positive effect on secondary production as enrichment with a limiting resource.

## Materials and Methods

### Overview

The main purpose of this study was to test the hypothesis that increased producer diversity can have similar (positive) effects on primary production and herbivore performance as has enrichment with light. To do so, we first reanalyzed data from two earlier experiments in which we manipulated light supply and producer diversity separately and in absence of grazers. We then described the grazer experiment, in which we manipulated light supply and algal species richness in a full factorial design in presence of a generalist grazer, the cladoceran *Daphnia magna*. As we were solely interested in assessing (and comparing) conjectured *positive* effects of enrichment and producer diversity on grazers, we tried to avoid confounding negative effects of these factors. In particular, we excluded high light intensities (which could lead to unfavorably high C:P ratios, and thus low food quality, of algal biomass) and we excluded algal taxa known to be toxic or inedible. Light supply was therefore constrained to ≤120 *μ*mol photons PAR m^−2^ sec^−1^ and the algal species pool consisted exclusively of chlorophytes of similar size.

### Experiments without grazers

Striebel et al. (2009b) measured short-term primary production and longer term biomass accrual of nine species of chlorophytes as a function of light supply. Methodological aspects of this experiment are very similar to the grazer experiment described below and are specified in detail in the original publication. Important features concerning design, replication, duration, and environmental conditions are also listed in Table 1. Seven of the nine chlorophyte species are shared with the grazer experiment and three of the light treatments cover a similar range of light supplies (Table 1). For the purpose of this article, we have therefore reanalyzed the effects of light supply on biomass accrual and the C:P ratio of these seven chlorophytes over the light supply range 10–110 *μ*mol photons PAR m^−2^ sec^−1^.

**Table 1:** Comparison of treatment characteristics and environmental conditions in the reported experiments

	Striebel et al. (2009b)	Behl et al. (2011)	This study
Light treatments (*μ*mol quanta PAR m^−2^ sec^−1^)	10, 20, 110	90	30, 60, 90, 120
Species richness treatments	1	1, 2, 3, 4	1, 2, 4, 8
Number of taxa in species pool	7	9	11
Phosphorus in culture medium (*μ*g P L^−1^)	10	31	15
Culture volume (mL)	250	400	500
Duration (days)	14	21	11
Average medium exchange rate (% day^−1^)	10	12.5	3
Total number of replicates	63	24	80
Initial algal biovolume (*μ*m^3^ mL^−1^)	2.0	5.3	2.6
Temperature (°C)	20	20	20

Behl et al. (2011) measured biomass accrual of nine species of chlorophytes as a function of species richness. Methodological aspects of this experiment are, again, very similar to the grazer experiment described below and are specified in the original publication, the most important features being listed in Table 1. All nine chlorophyte species are shared with the grazer experiment. Behl et al. (2011) analyzed the effects of chlorophyte diversity on response parameters using Shannon diversity, whereas the grazer experiment was analyzed with species richness as the independent variable (see below). For the purpose of this article, we have therefore reanalyzed the data from Behl et al. (2011) based on species richness (range 1–4 chlorophyte taxa, Table 1).

### Grazer experiment

We used 11 different strains of freshwater chlorophytes of similar edibility and size (Table 2). The strains originated from the SAG Culture Collection of Algae (Göttingen) and were precultured for several weeks under constant conditions in a freshwater medium (COMBO; 15.0 *μ*g phosphorus L^−1^) appropriate for phytoplankton and zooplankton cultivation. We established a species diversity gradient with four diversity levels ranging from mono- to 8-spp. polycultures (1, 2, 4, and 8 different species). Each diversity level (except for the 11 monocultures) was replicated three times with different species compositions (no identical replicates), resulting in a total of 20 communities, randomly comprised of members from the species pool. We established a light intensity gradient with 30, 60, 90, and 120 *μ*mol quanta m^−2^ sec^−1^ (measured with a LI-COR LI 191SA Quantum Sensor, Lincoln, Nebraska in front of the experimental units). This light intensity gradient is within the typical range experienced by phytoplankton in the mixed layer of a temperate lake. The two gradients (light and diversity) were fully cross-classified, yielding a total of 80 treatments.

**Table 2:** Chlorophyte species used in monoculture and polycultures experiments and their mean biovolumes and cell sizes

Chlorophyte species	Maximum cell diameter (*μ*m)	Mean cell biovolume (*μ*m^3^)	In polyculture
*Chlamydomonas reinhardtii*	10.4	385.6	4b; 8a,c
*Monoraphidium minutum*	6.7	104.5	4a; 8a,b,c
*Scenedesmus obliquus*	17.7	294.8	4a,c; 8a,b,c
*Selenastrum capricornutum*	9.5	113.8	4a,c; 8a,b
*Desmodesmus subspicatus*	8.6	162.2	2c; 4b; 8a,b,c
*Golenkinia brevispicula*	11.9	907.9	2a; 4b; 8a
*Haematococcus pluvialis*	16.5	1203.0	2c; 4c; 8b,c
*Staurastrum tetracerum*	35.0	1641.0	8a,b,c
*Tetraedron minimum*	8.7	315.3	2b; 4c; 8b,c
*Crucigenia tetrapedia*	7.1	150.5	2b; 4a; 8a,b
*Pediastrum simplex* (single cells)	17.1	1125.4	2a; 4b; 8c

Polyculture labels refer to the number of species per community (2, 4, 8) and a letter code (a, b, c) identifying each of the three different communities per diversity treatment.

All treatments were inoculated with an identical total algal biovolume (2.62 × 10^6^ *μ*m³ mL^−1^ equaling 0.5 mg particulate organic carbon, POC, L^−1^), and different species contributed equal biovolumes to communities with two or more species. All inocula were grown under dim light conditions for 1 day before each treatment received a founder population of eight age-synchronized neonate *Daphnia magna* (maximum 12 hours after birth) from our laboratory stock. The communities (500 mL) were exposed to the experimental treatments in 650-mL cell culture flasks over an 11-day period, with a 10% medium exchange on days 3, 5, and 9. Temperature was constant at 20 ± 0.5°C with a 16-h-light/8-h-dark photoperiod regime. All communities were gently shaken twice a day to prevent algae from sinking and accumulating at the bottom of the culture flask. *Daphnia* populations were monitored qualitatively on a daily basis to follow reproduction and mortality events.

We sampled each algal community on day 1 (before adding the neonates), day 6, and day 11 (after removing all daphnids). Samples were poured through an 80-*μ*m mesh net to retain daphnids, exuviae, and large detrital particles. As a measure of algal biomass, POC was determined after filtration onto precombusted and acid-washed glass-fiber filters (Whatman GF/C, Whatman International Ltd, Kent, U.K.) by elemental analysis (Elemental Analyzer, EA 1110 CHNS, CE Instruments, Wigan, U.K.). Particulate phosphorus (PP) was measured after sulfuric acid digestion followed by molybdate reaction. Algal biomass C:P ratios (more precisely “seston C:P ratios”) were calculated as the molar ratio of POC:PP. (Strictly speaking, POC and PP values include, beside living algal biomass, all kinds of particulate matter, which are retained on the GF/C filter, e.g., large bacterial colonies. To be precise, we will use the term “seston” instead of “algae” throughout the manuscript, where it is appropriate.) Additionally, we fixed an aliquot of each sample with Lugol's iodine to determine initial (day 1) and final (day 11) phytoplankton composition by inverted microscopy using Utermöhl chambers. A minimum of 100 cells of every species was counted by scanning at least five perpendicular transects or 20 randomly distributed, distinct fields. AnalySIS software (Pro 2.11.006, Soft Imaging Software GmbH) was used to determine biovolumes of cells by measuring two-dimensional live pictures; biovolumes were calculated from geometric shapes according to Hillebrand et al. (1999) or our own adjustments.

*Daphnia* body lengths were measured at the onset of the experiment (50 neonates not used in the experiment) and at the end (day 11, all surviving founder individuals and juveniles that hatched during the experiment). Length measurements were obtained electronically employing a microscope combined with a video system (ALTRA_20_ Soft Imaging System) and cell^P^ software (Olympus Soft Imaging Solutions GmbH, Germany). Body length was defined as the distance from the upper edge of the compound eye to the base of the apical spine. Individual dry mass was calculated using the empirical length–mass relationship W = 11.824 × L^2.236^, where W is dry mass (*μ*g), and L is body length (mm) (Stibor 1995). On day 4, the day we first detected females with eggs, all founder individuals were scanned for eggs in their brood chambers, and the number of gravid females was determined.

### Data processing and statistics

Effects of light supply and algal species richness on response variables were analyzed with simple (experiments without grazers) or multiple (grazer experiment) linear regression on log transformed data (Table 3). As single data values cannot easily be determined in multiple linear regression plots, the same data are shown also as 2D linear regression plots together with their respective statistics (Figs. S1–S3, online appendix). When response variables included zero values, data were log(x+n) transformed, where n is the smallest detectable unit. Thus, *n* = 1 in case of the numbers of gravid and surviving founder *Daphnia* individuals, and *n* = average biomass of an individual *Daphnia* in case of final *Daphnia* biomass. Algal variables (biomass and C:P ratio) were averaged over days 6 and 11 to better reflect average food conditions for *Daphnia* (separate analyses of days 6 and 11 did, however, reveal qualitatively similar patterns). All statistical analyses were performed with SigmaPlot 11.0 (2008), Systat Software, Inc. Daphnids suffered complete mortality in 14 of the 80 communities. We included these communities in the statistical analyses of *Daphnia* responses, but excluded them from the analyses of algal responses. Results of algal statistics were, however, very similar whether those communities were included or excluded. To test for interactive effects of light enrichment and species richness on response variables, we additionally performed two-way ANOVAs on all data from the grazer experiment. None of the interactions were statistically significant (*n* = 66 or 80, all *P* > 0.23). We, therefore, do not report the ANOVA statistics in the results section.

**Table 3:** Simple and multiple linear regression statistics (log *y* = *a* + *b* × log SR + *c* × log Light) describing the influence of algal species richness (SR) and light intensity (Light) treatments on several independent algal and *Daphnia* response variables (*y*)

			Overall regression			Coefficients (*b, c*)				
					
*y*		*n*	*r^2^*	*p*	*a*	Log SR (SEM)	*P*	Log Light (SEM)	*P*	Ratio of (SPRC_SR_/SPRC_Light_)
Striebel et al. (2009b)										
a	Log algal biomass	61	0.30		2.64			0.37 (0.07)	***	
b	Log seston molar C:P ratio	61	0.22		1.97			0.42 (0.10)	***	
Behl et al. (2011)										
c	Log algal biomass	24	0.53		3.94	0.11 (0.02)	***			
d	Log biovol.-specific absorbance	24	0.29		−6.09	0.51 (0.17)	**			
e	Log POC-specific absorbance	24	0.11		0.89	0.25 (0.15)	n.s.			
This study										
f	Log algal biomass d 6&11	66	0.28	***	1.37	0.26 (0.11)	*	0.81 (0.19)	***	0.53
g	Log seston molar C:P ratio d 6&11	66	0.26	***	0.69	0.29 (0.12)	*	0.83 (0.20)	***	0.58
h	Log No. of surviving founders	80	0.26	***	−0.51	0.37 (0.10)	***	0.58 (0.16)	***	0.98
i	Log No. of gravid founders (day 4)	80	0.23	***	−0.41	0.18 (0.05)	***	0.24 (0.08)	**	1.15
j	Log *Daphnia* biomass (founders)	80	0.32	***	1.34	0.45 (0.10)	***	0.68 (0.16)	***	0.99
k	Log *Daphnia* biomass (juveniles)	80	0.28	***	−0.39	0.86 (0.21)	***	1.24 (0.32)	***	1.06
l	Log founders relative yield	31	0.42		−0.25	0.62 (0.13)	***			
m	Log juveniles relative yield	28	0.05		−0.06	0.30 (0.26)	n.s.			

Algal and *Daphnia* biomass (*μ*g POC L^−1^), seston C:P (atomic ratios); Light, PAR intensity (*μ*mol quanta m^−2^sec^−1^); d 6&11, mean of days 6 and 11; *n*, number of replicates; n.s., not significant; SPRC, standard partial regression coefficient; SEM, standard error of the mean.

* *P* < 0.05.

** *P* < 0.01.

*** *P* < 0.001.

In the grazer experiment, standardized partial regression coefficients (SPRCs) were used as a measure of the relative contributions of light supply and species richness to the response variables. SPRC was calculated as


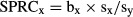


where b is the regression coefficient of the independent variable x (light or species richness), and s is the standard error of the independent (x) and dependent (y) variables, as determined in the multiple regression (Table 3). The relative contributions of light supply and species richness to a response variable was calculated as the ratio SPRC_SR_/SPRC_Light_, where ratios >1 indicate a larger relative contribution of species richness (SR) and ratios <1 indicate a larger relative contribution of light supply.

To further explore whether effects of algal species richness propagated to the herbivore level, we calculated the relative biomass yield of *Daphnia* as the ratio of observed *Daphnia* biomass in a the given polyculture *P*_*i*_ to the *Daphnia* biomass expected from monocultures of the algal species contributing to polyculture *P*_*i*_ as


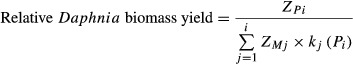


where *Z*_*Pi*_ is *Daphnia* biomass in polyculture *P*_*i*_ with *i* algal species, *Z*_*Mj*_ is *Daphnia* biomass in monoculture of algal species *j*, and *k*_*j*_(*P*_*i*_) = 1/*i* is the proportional contribution of algal species *j* to total algal biomass in polyculture *P*_*i*_ at the start of the experiment. For statistical analyses, relative yield was log transformed. Thus, overyielding occurred when the log transformed ratio was positive, and underyielding when it was negative. The occurrence of zero values (no surviving *Daphnia*) was addressed in two ways: either by excluding zero values from the analysis or by addition of the average biomass of one individual *Daphnia* to the values of *Z*_*Pi*_ and *Z*_*Mj*_ prior to log transformation. Results were similar and we only report the ones where zero values were excluded from the analysis.

## Results

### Experiments without grazers

**Figure 1:**
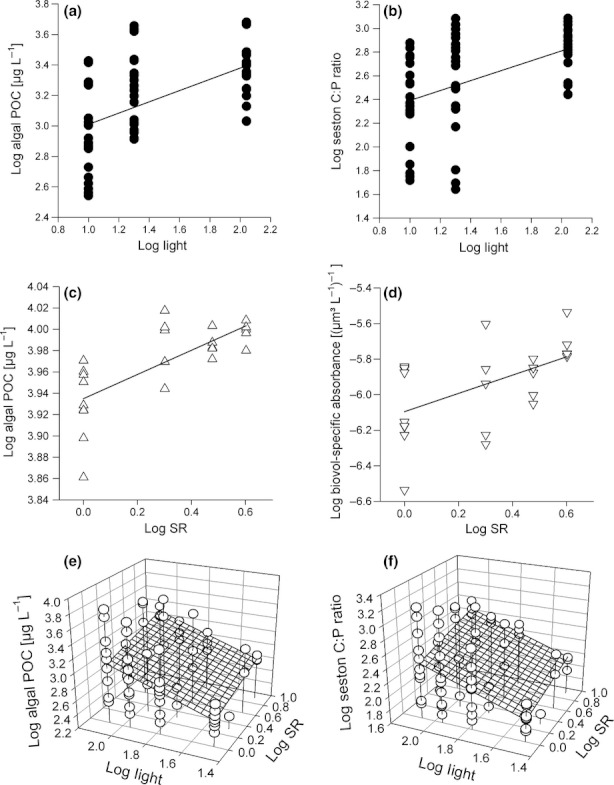
Influence of light intensity (Light) (*μ*mol quanta m^−2^sec^−1^) and/or phytoplankton species richness (SR) on (a) final algal biomass (*μ*g POC L^−1^) and (b) the final molar seston C:P ratio in the study by Striebel et al. (2009b), on (c) final biomass and (d) biovolume-specific PAR absorbance of chlorophytes in the study by Behl et al. (2011), and on (e) mean algal biomass and (f) mean molar seston C:P ratio on days 6 and 11 in the grazer experiment. All axes are log_10_ transformed. Linear regression equations and statistics are given in Table 3.

In absence of grazers, final algal biomass and the algal C:P ratio were both positively related to light supply over the range 10–110 *μ*mol quanta m^−2^ sec^−1^ (Fig. 1a and b; Table 3a, b). Final algal biomass was also positively related to species richness over the investigated gradient from 1 to 4 chlorophyte taxa (Fig. 1c; Table 3c). Species richness had furthermore a positive effect on specific PAR absorbance per unit algal biovolume (Fig. 1d; Table 3d) and per unit algal POC, the latter being nonsignificant (Table 3e; see Behl et al. 2011 for a description of how absorbance was measured).

### Grazer experiment

All phytoplankton monocultures and species mixtures established as scheduled, with proportions of component algal species on day 1 deviating in polycultures by only 10 ± 1% (mean±SEM) from the scheduled equal biovolumes. By the end of the experiment, algal species richness had not changed in any community, but evenness had decreased notably in all communities (see Table S1, online appendix). In 14 of the 80 communities, daphnids suffered complete mortality. This happened primarily in low-light and low-diversity communities. One algal monoculture (*Staurastrum tetracerum*) did not support positive *Daphnia* growth rates at any light level.

Both algal species richness and light intensity had positive effects on algal biomass and the seston C:P ratio averaged over days 6 and 11 (Fig. 1e and f and Table 3f, g), the effect of species richness being somewhat weaker than the effect of light (ratio SPRC_SR_/SPRC_Light_ 0.53–0.58).

**Figure 2:**
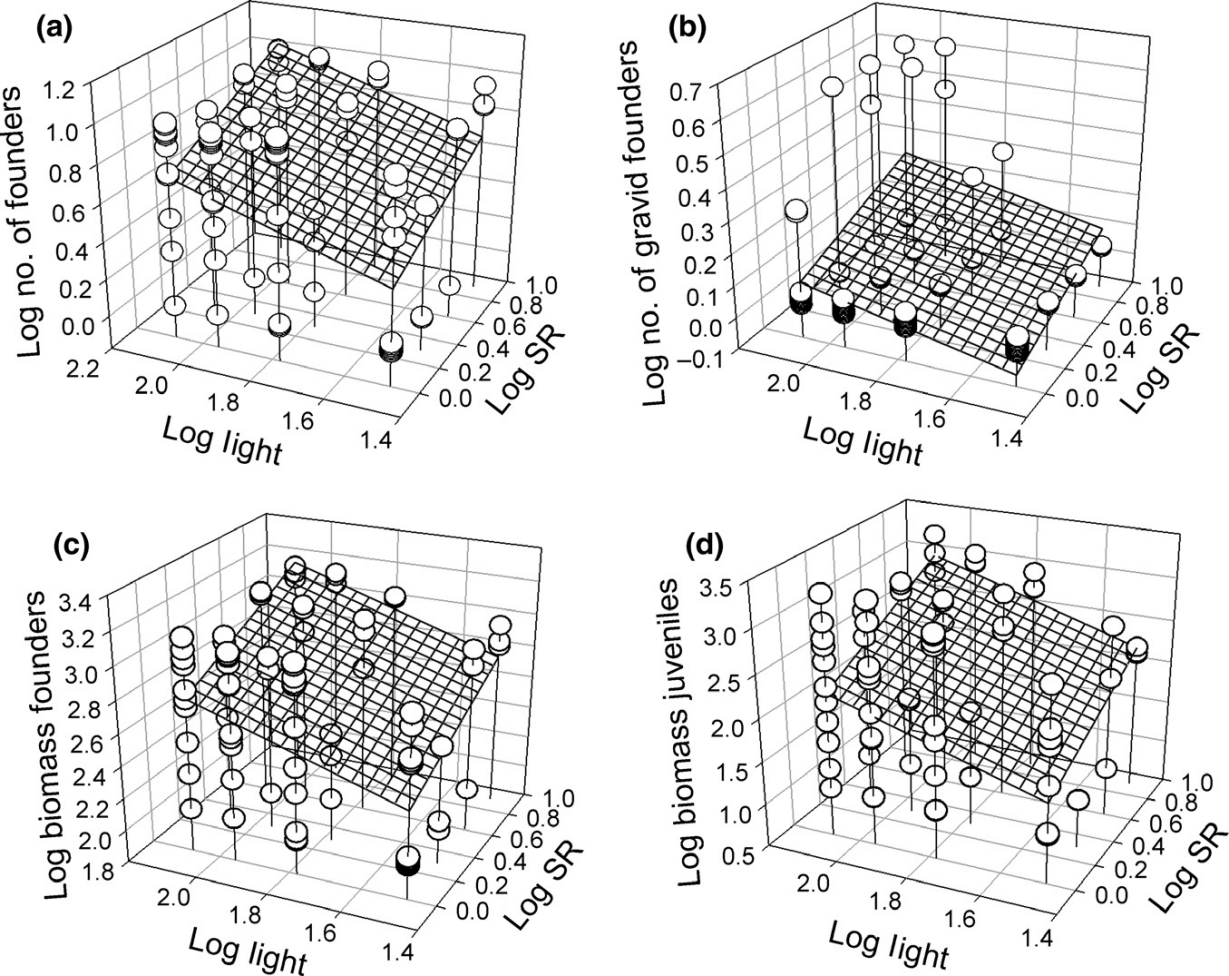
Influence of light intensity (Light) (*μ*mol quanta m^−2^ sec^−1^) and phytoplankton species richness (SR) on (a) the number of *Daphnia* founder individuals surviving to the end of the experiment, (b) the number of founder individuals carrying eggs in their brood chambers on day 4, and on the biomasses (*μ*g POC L^−1^) of (c) founder individuals and (d) juvenile *Daphnia* at the end of the experiment. All axes are log_10_ transformed. Replicate treatments with identical *y*-axis values have been slightly offset to make them visible. Multiple linear regression equations and statistics are given in Table 3.

Both algal species richness and light intensity had positive effects on the number of surviving founder individuals (Fig. 2a, Table 3h). Similarly, algal species richness and light intensity had positive effects on the number of egg-carrying individuals on day 4 (Fig. 2b; Table 3i). Note that on day 4, most daphnids did not yet carry eggs in their brood chambers (egg-carrying individuals occurred only in 15% of the populations).

**Figure 3:**
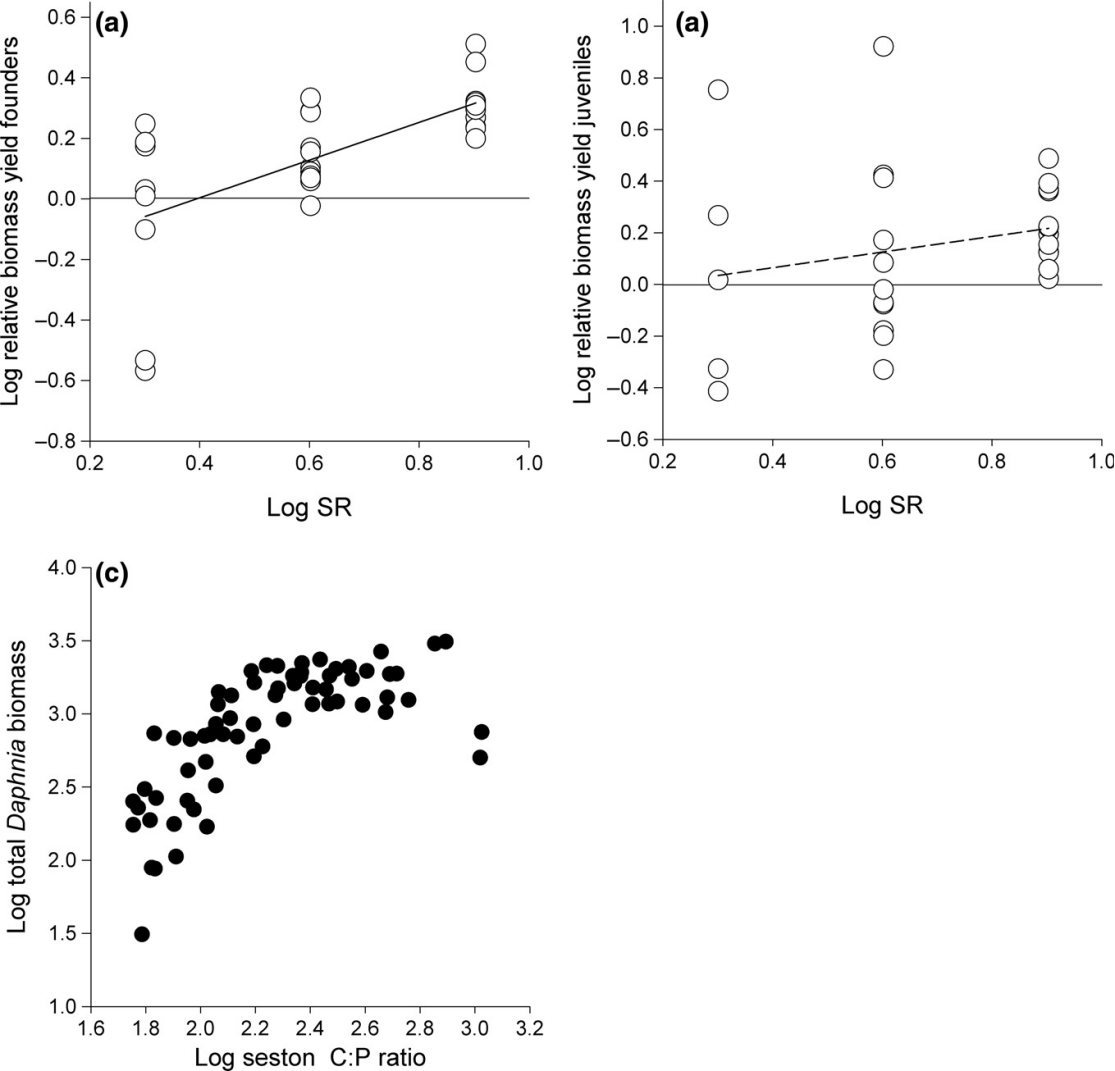
Influence of phytoplankton species richness (SR) on the relative biomass yield of (a) founder individuals and (b) juvenile *Daphnia*. Values >0 indicate overyielding, the means being significantly >0 in both cases (*t*-test). Linear regression equations and statistics are given in Table 3. (c) Relationship between the seston C:P ratio (mean of days 6 and 11) and final total *Daphnia* biomass (*μ*g POC L^−1^) (founders plus juveniles). The positive correlation levels off at a seston C:P ratio of c. 250. All axes are log_10_ transformed.

Algal species richness and light intensity had positive effects on the biomasses of both surviving founder individuals and of juvenile daphnids (Fig. 2c and d, Table 3j, k). A positive effect of algal species richness on *Daphnia* biomass was also supported by the calculations of relative biomass yield. The log of this ratio was on average 0.15 (and significantly larger than zero, *t*-test) for both founders and juveniles (Fig. 3a and b), corresponding to an untransformed relative biomass yield of 1.16 and, thus, on average 16% higher *Daphnia* biomass in polycultures compared with monocultures. In addition, the relative *Daphnia* biomass yield was positively related to algal species richness, the relationship being statistically significant for founders, but not for juveniles (Fig. 3a and b; Table 3l, m).

For all *Daphnia* response variables, the ratio SPRC_SR_/SPRC_Light_ was close to 1 (Table 3h–k), indicating that the positive effects of algal species richness and light on *Daphnia* performance were quantitatively very similar.

### Impact of algal species identity in polycultures

Notable differences in *Daphnia* biomass were observed in phytoplankton monocultures (see Fig. 2c and d). Monocultures of *Scenedesmus obliquus* yielded on average the highest final grazer biomasses of all monocultures (all light levels; Mann–Whitney Rank Sum Test; *U* = 16.0, *P* = 0.009, *n* = 44), and *Scenedesmus* exhibited on average the highest final proportion (49%) of all algal species in polycultures where it was present. However, no relationship between *Daphnia* biomass and the final proportion of *Scenedesmus* in these polycultures could be detected (linear regression: *r*^2^ = 0.03, *P* = 0.41, *n* = 21, data not shown). There was also no significantly positive relationship between *Daphnia* biomass and the final proportion of any other algal species in polyculture (see Fig. S4, online appendix). This fits well with the results from the experiments without grazers, where the positive effect of species richness on chlorophyte biomass was exclusively related to complementarity, the mean selection effect being zero (Behl et al. 2011).

## Discussion

### Functional equivalence of biodiversity and resource enrichment effects

We have investigated the hypothesis that positive effects of producer diversity on primary production are transferred up the food chain, and that these effects may be comparable to effects of enrichment with a production limiting resource. Our point of departure was the recent experimental demonstration that primary production is positively related to the taxonomic diversity of microalgae, and that this relationship is largely a consequence of overyielding (i.e., higher community production than expected based on the yields of the constituent species in monoculture; Striebel et al. 2009a; Behl et al. 2011; Cardinale 2011). Overyielding, in turn, is a strong indication of niche complementarity, and at least two complementarity mechanisms have been described for microalgae: spectral complementarity with respect to the capture of photons (Stomp et al. 2004; Striebel et al. 2009a; Behl et al. 2011), and hydraulic complementarity with respect to nutrient uptake in heterogeneous flow environments (Cardinale 2011). Niche complementarity has been documented also among terrestrial primary producers (e.g., Fridley 2003; van Ruijven and Berendse 2005). From the perspective of an herbivore population, increasing producer diversity can therefore be interpreted as just another form of enrichment: increasing the availability of resources that limit primary production, or increasing the producer community's ability to exploit those resources (through increased trait diversity), can have qualitatively similar effects on primary production, which, in turn, should be similarly transferred to herbivores.

The combined evidence from our experiments supports this hypothesized functional equivalence of resource availability and resource exploitation capacity: primary production, algal nutrient use efficiency (measured as algal C:P), and survival, reproduction, and biomass of *Daphnia* were all positively related to both light supply and producer diversity. Relative grazer yield was higher in poly- than in monocultures, compatible with the hypothesis that the positive effects of algal diversity on *Daphnia* were a consequence of niche complementarity at the producer level. The positive relationship between algal species richness and specific PAR absorbance per algal biomass is furthermore in line with Striebel et al.'s (2009a) observation of spectral complementarity in diverse algal communities. Consequently, more diverse algal communities may have exploited the PAR spectrum more efficiently because of a greater diversity of photosynthetic pigments. Re-analyzing the data for the nine chlorophytes species studied by Behl et al. (2011), we found indeed a positive relationship between species richness and pigment richness (data not shown). This relationship was, however, not statistically significant (*r*^2^ = 0.08, *P* = 0.18), possibly because only eight different pigments were distinguished (compared with 26 pigments in Striebel et al. 2009a).

The effects of light enrichment and algal diversity on grazers were quantitatively similar over the investigated range of treatment conditions, as indicated by equal magnitudes of their standard partial regression coefficients. On average, the addition of one species to the algal community had roughly the same positive effect on *Daphnia* biomass as had light enrichment by 14 *μ*mol quanta m^−2^ sec^−1^. While these numbers cannot be extrapolated to wider ranging field conditions (positive effects on algal production are, for example, expected to saturate at higher levels of both light supply and species richness), our experiments clearly illustrate that biodiversity effects can be transferred up the food chain just as easily as “traditional” enrichment effects and may be quantitatively equally important.

### Herbivore responses to producer diversity in other studies

Gamfeldt and coworkers reported on a producer–consumer experiment, where ciliate diversity had a strong negative effect on total algal biomass, and algal diversity had a strong positive effect on total consumer biomass (Gamfeldt et al. 2005). By contrast, a long-term (60 days) laboratory experiment conducted by Fox (2004) did not reveal any positive effects of ciliate diversity on their resource (algae) uptake and biomass accumulation. Notably, both experiments did not find a clear positive effect of algal diversity on primary production in the absence of grazers. Several earlier experimental studies, which documented positive effects of plant diversity on primary production, did also report herbivore responses. These studies found positive (Pfisterer et al. 2003), negative (Koricheva et al. 2000), or no responses (Siemann et al. 1998) of herbivore abundance to plant diversity manipulations. In all of these studies, however, experimental communities included multiple herbivores and higher level consumers and parasitoids. This makes it impossible to distinguish effects of plant productivity from community feedbacks within and above the herbivore level. Moreover, none of the studies did simultaneously investigate the effects of resource enrichment. Consequently, these studies cannot address our hypothesis that effects of niche complementarity on primary production can be transferred up the food chain. We are aware of only a single study using a relevant design (Steiner 2001); this study found temporally shifting responses, which will be discussed further down.

### Algal nutrient use efficiency and food quality

Similar to experiments with natural lake communities (Dickman et al. 2006; Striebel et al. 2008), seston C:P ratios increased with both increasing light supply and species richness. We have interpreted higher seston C:P ratios as an indication of increased algal nutrient use efficiency that made more energy available to grazers. At very high algal C:P ratios, the phosphorus content in algal biomass may, however, become so diluted that *Daphnia* growth is increasingly phosphorus rather than energy limited (Sterner 1993). Under such circumstances, further light enrichment can decrease *Daphnia* performance (Urabe and Sterner 1996). The phosphorus and light supplies in our experiment were deliberately chosen to represent moderate regimes. Nevertheless, 33% of all treatments had seston C:P ratios >250, and a plot of *Daphnia* biomass against seston C:P reveals that the otherwise strong positive correlation between the two variables leveled off around this threshold value (Fig. 3c). Our data thus suggest that the positive effects of both light enrichment and producer diversity on grazers may saturate and even turn negative at high levels of nutrient use efficiency (=high carbon-to-nutrient ratios of producer biomass). A truly negative influence of seston C:P on *Daphnia* performance, which could have obscured the positive effects of light enrichment and algal diversity, is, however, only indicated for the two treatments with the highest seston C:P ratios exceeding 1000 (Fig. 3c).

### Other prey diversity effects on grazers: diet mixing and prey defenses

Although our experiments are clearly consistent with the hypothesis that the positive effects of producer diversity on grazer performance were mediated by increased algal production, it is possible that a second form of producer complementarity contributed to this effect, that is, dietary mixing. The biochemical composition of autotrophs is typically imbalanced with respect to the nutritional needs of their herbivores (Elser et al. 2000; Sperfeld et al. 2012). Consequently, any single plant species will often be deficient in some essential biochemical compounds, and there is growing evidence that herbivores regulate their intake of such compounds by mixing nutritionally complementary plants in their diets (Simpson et al. 2004). Diet mixing has indeed been shown to improve performance in a wide range of herbivores including mammals, fish, and grasshoppers (Pennings et al. 1993; Unsicker et al. 2008; Wang et al. 2010).

Positive effects of diet mixing have also been documented in *Daphnia* and other cladocerans (Boersma and Vijverberg 1995; DeMott 1998), but the evidence is rather mixed (e.g., Narvani and Mazumder 2010). Most cladocerans, including *Daphnia*, are filter feeders with very limited ability to actively select or avoid specific particles. Consequently, *Daphnia* performance is typically reduced when unpalatable or toxic species are present in a food mixture (Gliwicz and Lampert 1990; Lürling 2003). Increased algal diversity could therefore have negative effects on grazers, if unpalatable or toxic taxa are common in the algal species pool. We tried to avoid this issue by only including nontoxic, unicellular chlorophytes in the edible size range in the species pool. With the exception of *Staurastrum*, all algal taxa did indeed support *Daphnia* populations when grown in monoculture (at least at some levels of light enrichment), indicating that poor algal food quality was a minor issue in our experiment. Also, total *Daphnia* biomass accrual in polycultures was independent of the monoculture yields, suggesting that *Daphnia* performance was not driven by the relative abundance of particularly “good” (e.g., *Scenedesmus*) or “poor” (e.g., *Staurastrum*) food algae (see Fig. S4, online appendix).

From the reverse perspective, low food quality did also not seem to convey a grazer-mediated competitive advantage to algae. If anything, the opposite was observed. The species yielding the highest *Daphnia* biomass in monoculture (*Scenedesmus*) was also most successful in polycultures (averaging 49% of total final algal biovolume), whereas *Staurastrum* did poorly in most polycultures (averaging 2.5% of total final algal biovolume), although it did not support *Daphnia* growth in monoculture. The latter observation is interesting given the predominant discussion of negative effects of producer diversity on herbivory in the literature (Hillebrand and Cardinale 2004). In particular, it has been argued that more diverse prey communities are more likely to include unpalatable, toxic, or inedible species that would increase in frequency under grazing pressure and ultimately dominate the community (Duffy et al. 2007). This phenomenon has indeed been reported from natural and artificially assembled communities (Steiner 2001; Fox 2004; Edwards et al. 2010). However, other experiments show contrasting results in favor of the “balanced diet hypothesis” (Duffy et al. 2007), which states that consumers benefit from a more diverse prey community, due to broader availability of qualitatively different food resources.

### Interactive effects of enrichment and producer diversity

We are aware of only one other study with a somewhat similar experimental design as ours. Steiner (2001) investigated the transfer of primary production to herbivores as a function of producer diversity and nutrient enrichment. Specifically, he created different plankton communities by adding a single grazer species (*Daphnia pulex* or *Ceriodaphnia quadrangula*) to either a monoculture of an edible green alga or a mix of the green alga with a diverse community of pond phytoplankton. The two phytoplankton diversity treatments were cross-classified with two levels of nutrient enrichment. Similar to our experiment, grazer biomass was positively affected by both enrichment and producer diversity during the first 21 days of the experiment. The positive diversity effect on grazers switched, however, sign to a negative one later in the experiment (days 28–42), when grazing resistant algal taxa came to dominate in high-diversity treatments. This reversal was, however, only observed in the nutrient enriched treatments, which is consistent with theory. Assuming a resource competition-grazing resistance tradeoff in producers, theory predicts that grazer biomass increases strongly with enrichment when grazing resistant taxa are absent from the community, but only weakly, so when grazing resistant taxa are present; grazing resistant taxa, in turn, are predicted to be competitively excluded at low levels of enrichment (Holt et al. 1994; Grover 1995).

Clearly, more experimental studies are needed to clarify the conditions under which increased producer diversity enhances trophic transfer of primary production up the food chain. Most importantly, results from simple and semi-artificial laboratory food webs need to be confronted with “real world scenarios.” Although eco-physiological mechanisms that underlie diversity effects (e.g., complementary resource use among producers) can often only be explored in a controlled and thus simplified environment, one has to keep in mind that in natural communities (i.e., on larger scales in space, time, and complexity), trophic effect magnitudes might be much more constrained by feedback mechanisms such as the promotion of inedible species or predation on grazers. A comparison of Steiner's (2001) results with ours tentatively suggests that positive effects of producer diversity on grazers might be transient, if grazing resistant taxa are present in the species pool and if the system is sufficiently enriched. Conversely, if limiting resources are scarce or if grazing resistant taxa are absent, positive effects of producer diversity on grazers should persist also in the long run. In a long-term experiment using a similar pool of edible algal species as this study, positive effects of producer diversity on *Daphnia* were indeed observed over hundreds of days (Behl 2012). It should also be kept in mind that even when positive diversity effects are transient, they may nevertheless be ecologically highly relevant. For example, many plankton communities go through recurrent periods of transient dynamics driven by seasonality and disturbances (Sommer et al. 1986), and in many systems, a large fraction of the transfer of primary production to higher trophic levels occurs during the transient population peaks, like the phytoplankton spring bloom and the subsequent grazer peak in temperate lakes (e.g., Platt et al. 2003; Winder and Schindler 2004; Berger et al. 2010; Klausmeier and Litchman 2012). We, therefore, propose that trophic transfer of biodiversity effects has the potential to affect both long-term and seasonal community dynamics and related ecosystem services, and that the concept needs to be included in future biodiversity research.

## Supporting Information

**Figure S1.** Influence of light intensity (Light) (µmol quanta m^−2^sec^−1^) or phytoplankton species richness (SR) on (a, b) mean algal biomass (µg POC L^−1^) and (c, d) mean molar seston C:P ratio on days 6 and 11 in the grazer experiment.

**Figure S2.** Influence of light intensity (Light) (µmol quanta m^−2^sec^−1^) or phytoplankton species richness (SR) on (a, b) the number of surviving founder daphnids and (c, d) the number of gravid founder daphnids on day 4.

**Figure S3.** Influence of light intensity (Light) (µmol quanta m^−2^sec^−1^) or phytoplankton species richness (SR) on (a, b) on the biomasses (µg POC L^−1^) of (c) founder individuals and (d) juvenile *Daphnia* at the end of the experiment.

**Figure S4**. Final total *Daphnia* biomass as a function of mean biovolume-based proportions of algal species in polycultures (closed circles) and monocultures (open circles).

**Tabel S1.** Final biovolume-based proportions of algal species in polycultures.
