# Supplementary material for: Trophic transfer of biodiversity effects: functional equivalence of prey diversity and enrichment?
Source: Ecol Evol. 2012 Nov 8;2(12):3110–22. doi: 10.1002/ece3.415 (PMC3539004; doi:10.1002/ece3.415)
Supplement: Supplementary file 1 [file ece30002-3110-SD1.docx]

**Figure S1:** Influence of light intensity (Light) [µmol quanta m^-2^s^-1^] or phytoplankton species richness (SR) on (a, b) mean algal biomass [µg POC L^-1^] and (c, d) mean molar seston C:P ratio on days 6 and 11 in the grazer experiment. All axes are log_10_ transformed. Linear regression equations and statistics are: a) Log algal POC = 2.88+0.25×Log SR, r² = 0.06, p = 0.05. b) Log algal POC = 1.47+0.81×Log Light, r² = 0.22, p < 0.0001. c) Log seston C:P ratio = 2.24+0.27×Log SR, r² = 0.06, p = 0.05. d) Log seston C:P ratio = 0.82+0.81×Log Light, r² = 0.19, p = 0.0002.
