# Supplementary material for: Trophic transfer of biodiversity effects: functional equivalence of prey diversity and enrichment?
Source: Ecol Evol. 2012 Nov 8;2(12):3110–22. doi: 10.1002/ece3.415 (PMC3539004; doi:10.1002/ece3.415)
Supplement: Supplementary file 2 [file ece30002-3110-SD2.docx]

**Figure S2**: Influence of light intensity (Light) [µmol quanta m^-2^s^-1^] or phytoplankton species richness (SR) on (a, b) the number of surviving founder daphnids and (c, d) the number of gravid founder daphnids on day 4. All axes are log_10_ transformed. Replicate treatments with identical y-axis values have been slightly offset to make them visible. Linear regression equations and statistics are: a) Log no. of founders = 0.55+0.38×Log SR, r² = 0.13, p = 0.0011. b) Log no. of founders = -0.40+0.58×Log Light, r² = 0.13, p = 0.001. c) Log no. of gravid founders = 0.05+0.16×Log SR, r² = 0.12, p = 0.0018. d) Log seston C:P ratio = -0.82+0.21×Log Light, r² = 0.08, p = 0.01.
