# Supplementary material for: Trophic transfer of biodiversity effects: functional equivalence of prey diversity and enrichment?
Source: Ecol Evol. 2012 Nov 8;2(12):3110–22. doi: 10.1002/ece3.415 (PMC3539004; doi:10.1002/ece3.415)
Supplement: Supplementary file 3 [file ece30002-3110-SD3.docx]

**Figure S3**: Influence of light intensity (Light) [µmol quanta m^-2^s^-1^] or phytoplankton species richness (SR) on (a, b) on the biomasses [µg POC L^-1^] of (c) founder individuals and (d) juvenile *Daphnia* at the end of the experiment. All axes are log_10_ transformed. Replicate treatments with identical y-axis values have been slightly offset to make them visible. Linear regression equations and statistics are: a) Log biomass founders = 2.58+0.45×Log SR, r² = 0.16, p = 0.0003. b) Log biomass founders = 1.42+0.70×Log Light, r² = 0.17, p = 0.002. c) Log biomass juveniles = 1.87+0.86×Log SR, r² = 0.15, p = 0.0004. d) Log biomass juveniles = -0.15+1.24×Log Light, r² = 0.14, p = 0.0007.
