# Supplementary material for: Trophic transfer of biodiversity effects: functional equivalence of prey diversity and enrichment?
Source: Ecol Evol. 2012 Nov 8;2(12):3110–22. doi: 10.1002/ece3.415 (PMC3539004; doi:10.1002/ece3.415)
Supplement: Supplementary file 4 [file ece30002-3110-SD4.docx]

**Figure S4**: Final total *Daphnia* biomass as a function of mean biovolume-based proportions of algal species in polycultures (closed circles) and monocultures (open circles). Algal mean proportions are calculated as arithmetic average of initial and final proportions. Algal species abbreviations: Chl: *Chlamydomonas reinhardtii*, Mon: *Monoraphidium minutum*; Sce: *Scenedesmus obliquus*; Sel: *Selenastrum capricornutum*; Des: *Desmodesmus subspicatus*; Gol: *Golenkinia brevispicula*; Hae: *Haematococcus pluvialis*; Sta: *Staurastrum tetracerum*; Tet: *Tetraedron minimum*; Cru: *Crucigenia tetrapedia*; Ped: *Pediastrum simplex*.
