# Supplementary material for: Trophic transfer of biodiversity effects: functional equivalence of prey diversity and enrichment?
Source: Ecol Evol. 2012 Nov 8;2(12):3110–22. doi: 10.1002/ece3.415 (PMC3539004; doi:10.1002/ece3.415)
Supplement: Supplementary file 5 [file ece30002-3110-SD5.docx]

**Tabel S1**: Final biovolume-based proportions of algal species in polycultures. Polyculture labels refer to the number of species (2, 4, 8) and to the three different communities per species richness level (a, b, c). Each community was exposed to four different light levels (30, 60, 90, 120 µmol quanta PAR m^-2^ s^-1^). Algal species abbreviations are: Chl: *Chlamydomonas reinhardtii*, Mon: *Monoraphidium minutum*; Sce: *Scenedesmus obliquus*; Sel: *Selenastrum capricornutum*; Des: *Desmodesmus subspicatus*; Gol: *Golenkinia brevispicula*; Hae: *Haematococcus pluvialis*; Sta: *Staurastrum tetracerum*; Tet: *Tetraedron minimum*; Cru: *Crucigenia tetrapedia*; Ped: *Pediastrum simplex*.

| Label | Light | Final proportions of algal species | | | | | | | | | | |
| --- | --- | --- | --- | --- | --- | --- | --- | --- | --- | --- | --- | --- |
|  |  | Chl | Mon | Sce | Sel | Des | Gol | Hae | Sta | Tet | Cru | Ped |
| 2-a | 30 |  |  |  |  |  | 0.23 |  |  |  |  | 0.77 |
|  | 60 |  |  |  |  |  | 0.12 |  |  |  |  | 0.88 |
|  | 90 |  |  |  |  |  | 0.07 |  |  |  |  | 0.93 |
|  | 120 |  |  |  |  |  | 0.05 |  |  |  |  | 0.95 |
| 2-b | 30 |  |  |  |  |  |  |  |  | 0.97 | 0.03 |  |
|  | 60 |  |  |  |  |  |  |  |  | 0.98 | 0.02 |  |
|  | 90 |  |  |  |  |  |  |  |  | 0.88 | 0.12 |  |
|  | 120 |  |  |  |  |  |  |  |  | 0.79 | 0.21 |  |
| 2-c | 30 |  |  |  |  | 0.73 |  | 0.27 |  |  |  |  |
|  | 60 |  |  |  |  | 0.38 |  | 0.62 |  |  |  |  |
|  | 90 |  |  |  |  | 0.42 |  | 0.58 |  |  |  |  |
|  | 120 |  |  |  |  | 0.94 |  | 0.06 |  |  |  |  |
| 4-a | 30 |  | 0.33 | 0.62 | 0.04 |  |  |  |  |  | 0.01 |  |
|  | 60 |  | 0.26 | 0.70 | 0.03 |  |  |  |  |  | 0.02 |  |
|  | 90 |  | 0.24 | 0.71 | 0.03 |  |  |  |  |  | 0.03 |  |
|  | 120 |  | 0.13 | 0.83 | 0.01 |  |  |  |  |  | 0.02 |  |
| 4-b | 30 | 0.76 |  |  |  | 0.16 | 0.03 |  |  |  |  | 0.05 |
|  | 60 | 0.89 |  |  |  | 0.09 | 0.01 |  |  |  |  | 0.01 |
|  | 90 | 0.86 |  |  |  | 0.07 | 0.04 |  |  |  |  | 0.03 |
|  | 120 | 0.91 |  |  |  | 0.01 | 0.03 |  |  |  |  | 0.06 |
| 4-c | 30 |  |  | 0.93 | 0.04 |  |  | 0.01 |  | 0.02 |  |  |
|  | 60 |  |  | 0.94 | 0.01 |  |  | 0.02 |  | 0.03 |  |  |
|  | 90 |  |  | 0.90 | 0.01 |  |  | 0.02 |  | 0.07 |  |  |
|  | 120 |  |  | 0.84 | 0.02 |  |  | 0.06 |  | 0.09 |  |  |
| 8-a | 30 | 0.01 | 0.73 | 0.21 | 0.01 | 0.03 | 0.01 |  | 0.01 |  | 0.02 |  |
|  | 60 | 0.03 | 0.82 | 0.11 | 0.01 | 0.01 | 0.01 |  | 0.02 |  | 0.01 |  |
|  | 90 | 0.13 | 0.64 | 0.15 | 0.02 | 0.04 | 0.03 |  | 0.01 |  | 0.01 |  |
|  | 120 | 0.18 | 0.25 | 0.48 | 0.01 | 0.01 | 0.02 |  | 0.02 |  | 0.03 |  |
| 8-b | 30 |  | 0.51 | 0.45 | 0.01 | 0.01 |  |  | 0.00 | 0.02 | 0.01 |  |
|  | 60 |  | 0.46 | 0.34 | 0.03 | 0.03 |  |  | 0.04 | 0.07 | 0.01 |  |
|  | 90 |  | 0.37 | 0.40 | 0.02 | 0.09 |  |  | 0.04 | 0.08 | 0.01 |  |
|  | 120 |  | 0.45 | 0.31 | 0.01 | 0.09 |  |  | 0.04 | 0.07 | 0.03 |  |
| 8-c | 30 | 0.05 | 0.43 | 0.43 |  | 0.02 |  | 0.01 | 0.01 | 0.03 |  | 0.01 |
|  | 60 | 0.06 | 0.66 | 0.12 |  | 0.02 |  | 0.05 | 0.03 | 0.04 |  | 0.01 |
|  | 90 | 0.02 | 0.57 | 0.24 |  | 0.01 |  | 0.09 | 0.02 | 0.01 |  | 0.04 |
|  | 120 | 0.05 | 0.59 | 0.19 |  | 0.05 |  | 0.01 | 0.06 | 0.03 |  | 0.02 |
